# Polymorphism analysis of propeller domain of *k13* gene in *Plasmodium ovale curtisi* and *Plasmodium ovale wallikeri* isolates original infection from Myanmar and Africa in Yunnan Province, China

**DOI:** 10.1186/s12936-020-03317-2

**Published:** 2020-07-13

**Authors:** Mengni Chen, Ying Dong, Yan Deng, Yanchun Xu, Yan Liu, Canglin Zhang, Herong Huang

**Affiliations:** 1 Yunnan Institute of Parasitic Diseases, Yunnan Provincial Key Laboratory of Vector-Borne Diseases Control and Research, Yunnan Centre of Malaria Research, Academician Workstation of Professor Jin Ningyi, Expert Workstation of Professor Jiang Lubin, Pu’er, 665000 China; 2 School of Basic Medical Sciences, Dali University, Dali, 667000 China

**Keywords:** Yunnan province, Imported, *P. ovale*, Haplotype, *k13* gene, Sequence, Polymorphism

## Abstract

**Background:**

Eighteen imported ovale malaria cases imported from Myanmar and various African countries have been reported in Yunnan Province, China from 2013 to 2018. All of them have been confirmed by morphological examination and 18S small subunit ribosomal RNA gene (18S rRNA) based PCR in YNRL. Nevertheless, the subtypes of *Plasmodium ovale* could not be identified based on 18S rRNA gene test, thus posing challenges on its accurate diagnosis. To help establish a more sensitive and specific method for the detection of *P. ovale* genes, this study performs sequence analysis on *k13*-propeller polymorphisms in *P. ovale.*

**Methods:**

Dried blood spots (DBS) from ovale malaria cases were collected from January 2013 to December 2018, and the infection sources were confirmed according to epidemiological investigation. DNA was extracted, and the coding region (from 206th aa to 725th aa) in *k13* gene propeller domain was amplified using nested PCR. Subsequently, the amplified products were sequenced and compared with reference sequence to obtain CDS. The haplotypes and mutation loci of the CDS were analysed, and the spatial structure of the amino acid peptide chain of *k13* gene propeller domain was predicted by SWISS-MODEL.

**Results:**

The coding region from 224th aa to 725th aa of *k13* gene from *P. ovale* in 83.3% of collected samples (15/18) were amplified. Three haplotypes were observed in 15 samples, and the values of Ka/Ks, nucleic acid diversity index (π) and expected heterozygosity (He) were 3.784, 0.0095, and 0.4250. *Curtisi* haplotype, *Wallikeri* haplotype, and mutant type accounted for 73.3% (11/15), 20.0% (3/15), and 6.7% (1/15). The predominant haplotypes of *P. ovale curtisi* were determined in all five Myanmar isolates. Of the ten African isolates, six were identified as *P. o. curtisi*, three were *P. o. wallikeri* and one was mutant type. Base substitutions between the sequences of *P. o. curtisi* and *P. o. wallikeri* were determined at 38 loci, such as c.711. Moreover, the A > ***T ***base substitution at c.1428 was a nonsynonymous mutation, resulting in amino acid variation of T476S in the 476th position. Compared with sequence of *P. o. wallikeri*, the double nonsynonymous mutations of G > ***A*** and A > ***T*** at the sites of c.1186 and c.1428 leads to the variations of D396N and T476S for the 396th and 476th amino acids positions. For *P. o. curtisi* and *P. o. wallikeri*, the peptide chains in the coding region from 224th aa to 725th aa of *k13* gene merely formed a monomeric spatial model, whereas the double-variant peptide chains of D396N and T476S formed homodimeric spatial model.

**Conclusion:**

The propeller domain of *k13* gene in the *P. ovale* isolates imported into Yunnan Province from Myanmar and Africa showed high differentiation. The sequences of Myanmar-imported isolates belong to *P. o. curtisi*, while the sequences of African isolates showed the sympatric distribution from *P. o. curtisi*, *P. o. wallikeri* and mutant isolates. The CDS with a double base substitution formed a dimeric spatial model to encode the peptide chain, which is completely different from the monomeric spatial structure to encode the peptide chain from *P. o. curtisi* and *P. o. wallikeri*.

## Background

The increase of imported ovale malaria cases are a cause of concern in non-endemic and malaria-free countries. For instance, Canada diagnosed 49 cases from 2006 to 2015 [1]; Spain reported 35 cases from 2005 to 2011 [2, 3]; the USA diagnosed 376 cases from 2012 to 2016 [4]. All the 109 ovale malaria diagnosed in Jiangsu Province of China between 2011 and 2014 originated from Africa [5]. In some malaria-endemic countries, the continuous application of control and preventive measures has also led to notable changes in epidemiological patterns of malaria. Over the past 20 years, malaria in Tanzania has evolved from a preponderance of falciparum malaria to an increase of malariae and ovale malaria [6]. Among the influencing factors of the increased incidence of ovale malaria, the diagnostic error due to excessive reliance on microscopy to identify species of malaria parasite could not be fully ruled out.

When using light microscopy for diagnosis, the morphology of *P. ovale* can easily be confused with *Plasmodium vivax* in [7]. In a study of mono-infected ovale malaria cases diagnosed and reported in Yunnan Province from 2013 to 2018, 94.7% (18/19) were initially misdiagnosed as vivax malaria by microscopic examination in the county-level laboratory. It was not until 1993, when Snounou et al*.* [8] developed a method for molecular identification of *Plasmodium* species by amplifying the 18S rRNA gene of the parasite using polymerase chain reaction (PCR), that an accurate identification of *Plasmodium* species became possible. Since then, human infections with *Plasmodium knowlesi* were confirmed by PCR [9, 10], and numerous dimorphisms in the locus of *P. ovale* genome were identified. This established the theory that at least 6 malaria parasites could infect humans, including *Plasmodium falciparum, Plasmodium malariae, P. vivax, P. knowlesi, P. ovale curtisi,* and *P. ovale wallikeri* [11–14]. Further analysis suggests that the distinction between *P. o. curtisi* and *P. o. wallikeri* is attributed to the fact that genetic recombination occurs only within 1 haplotype, rather than the accumulated long-term differentiation between 2 haplotypes [11, 12, 15].

Identifying the subtypes of *P. ovale* as *curtisi* and *wallikeri* subtype can help clinicians to predict the prognosis of individual ovale malaria patients after treatment. It is generally believed that *P. ovale curtisi* is more likely to relapse [16–19], while *wallikeri* subtype features a shorter incubation period [3, 16], with high incidence of thrombocytopenia and severe malaria.

Unfortunately, it has been found that the practicality of identifying *curtisi* subtype and *wallikeri* subtype based on the 18S rRNA gene dimorphism of *P. ovale* can be compromised by mutations in the 18S rRNA gene [20] or poly-chromosomal localization. Although the exact location of the 18S rRNA gene in the genome of *P. ovale* remains unclear, the copies of 18S rRNA gene of *P. vivax* and *P. falciparum* have been found on chromosomes 2, 3, 5, 6, 10, and 1, 5, 6, 11 (https://www.ncbi.nlm.nih.gov/gene), respectively. PCR amplification of 18S rRNA copies with inconsistent mapping may lead to wrong identification of species. Thus, scholars from many countries attempt to make up for the shortcomings with the single-gene dimorphism distinguish between *curtisi* subtype and *wallikeri* subtype by increasing the detection of target genes [7, 12]. For instance, the dihydrofolate reductase thymidylate synthase gene (*dhfr-ts*) and the tryptophan rich antigen gene (*Potra*), showed extensive synonymous and nonsynonymous polymorphisms between *P. o. curtisi* and *P. o. wallikeri* samples [7]. Nevertheless, some evaluated genes, such as *dhfr-ts,* were not widely used in distinguishing *P. ovale* subtype, probably due to the difficulty of amplification. In previous studies by Dong and the team, the proportion of amplified *dhfr-ts* gene in *P. vivax* isolates was only 25.8% (310/1203) [21]. In the current study, the feature that single copy of *k13* gene in the genome and the simplicity of intron-free insertion in the structure was used to provide reference for establishing another stable method for the detection and genotyping of *P. ovale* on the basis of revealing *k13* gene sequence dimorphism.

## Methods

### Ethics statement

The study was approved by Ethical Committee of Yunnan Institute of Parasitic Diseases. Genetic testing was performed on stored blood samples obtained as part of routine diagnostic work from febrile patients suspected of malaria. All samples were allocated unique code intead of any personal information, which will keep completely confidential and will not be disclosed to any individuals or organisations.

### Research subjects

The blood samples from ovale malaria patients, who were officially reported in Yunnan Province from January 2013 to December 2018 and registered by the China Information Management System for parasitic diseases control, were collected continuously. All blood samples on filter papers are air dried and properly restored for further examination. The mono-infection of *P. ovale* requires double parasitically confirmation by both microscopy and *Plasmodium* 18S rRNA gene detection by Yunnan Province Reference Laboratory (YNRL) (Additional file 1). The patients DBS were also used for the analysis of *k13* genetic polymorphism of *P. ovale* subtypes. The infection sources of ovale malaria cases were determined according to epidemiological investigation, i.e., those without a travel history to epidemic areas outside Yunnan Province within the last 30 days before the onset of malaria were defined as local cases; those who have a history of travelling to endemic regions, such as Myanmar and various countries in Africa, were regarded as imported cases [22, 23].

### Reagents

QIAamp DNA Mini Kit (QIAGEN Biotech, Germany), 2 × Taq PCR Mastermix (KT201, containing Taq enzyme) are purchased from QIAGEN Biotech (Hilden, Germany). Agarose and DNA markers were purchased from Takara Biotech (Dalian, China).

### Genomic DNA extraction

Three filter paper punches, each with a diameter of 5 mm, were taken, and *Plasmodium* genomic DNA was extracted according to the manufacturer’s instructions of the QIAamp DNA Mini Kit (QIAGEN Biotech, Germany), and the extracted DNA was stored at – 20 °C for later use.

### PCR amplification of the propeller domain in *k13* gene

Reference sequence with Accession No. LT594593.1 from GenBank (https://www.ncbi.nlm.nih.gov), no homology with other species, was used as template for design of primers and setting reaction conditions. The forward and reverse primers for first-round PCR used to amplify the coding region from 206th aa to 725th aa in *k13* gene were 5′-CGTGCCTATGAGAAAT-3′ and 5′-CATCTGCTTCGTCCA-3′, respectively, and the primers for the 2nd-round PCR were 5′-AACGGAGTTAAGTGATT-3′ and 5′-TGTATGGAGGGAAGG-3′, respectively. The expected fragments of the amplified product were 1991 bp for 1st-round PCR and 1732 bp for 2nd-round PCR, respectively. The reaction systems of the 2 round PCR(s) were: 2.6 µl of DNA template for the first round PCR reaction, 1.6 µl of first-round PCR product as template for the 2nd round PCR reaction, 14.0 µl of 2 × Taq PCR mix, 0.7 µl of upstream and downstream primers each (20 µM). The volume was increased to 25.0 µl with ddH_2_O. The PCR reaction conditions were: 94 °C for 3 min; 94 °C for 30 s, 49 °C for 90 s, 72 °C for 2 min, 35 cycles; 72 °C for 7 min in the first-round PCR and 94 °C for 3 min; 94 °C for 30 s, 59 °C for 90 s, 72 °C for 2 min, 35 cycles; 72 °C for 7 min for the second-round PCR. The second-round amplified products were observed on 1.5% agarose gel electrophoresis, and the positive products were sent to Shanghai Meiji Biomedical Technology Co., Ltd. for sequencing using the dideoxy chain-termination method.

### Alignment of the coding DNA sequence of propeller domain

The sequencing results were aligned using DNAStar 11.0 and BioEdit 7.2.5 software. All DNA sequences were assessed with the Basic Local Alignment Search Tool (BLAST, https://blast.ncbi.nlm.nih.gov/Blast.cgi) at NCBI platform in order to verify whether it belongs to *P. ovale* sequence. When DNA sequences were aligned with LT594593.1, these sequences with Identifications equals to 100% and the Query cover above 99%, were considered as *k13* gene sequence of *P. ovale*. The obtained DNA sequences were compared with *k13* gene *curtisi* subtype reference sequence (GenBank accession no. KT792971.1) [24] and *wallikeri* subtype reference sequence (GenBank accession no: KT792969.1) [24] to confirm the coding DNA sequence (CDS) in *k13* gene ranges (206th aa to 725th aa). We used MEGA 5.04 software to confirm nonsynonymous mutation and synonymous mutation sites in the CDS strand, and DnaSP 5.10 software to calculate the rate of nonsynonymous substitution (NSS, Ka), synonymous substitution (SS, Ks) and the value of Ka / Ks. Arlequin 3.01 software was used to analyze the haplotype of the CDS strand and to calculate the nucleic acid diversity index (π), the expected heterozygosity (He), and so forth [25].

### Spatial prediction of the peptide chain of *k13* gene

SWISS-MODEL (www.swissmodel.expasy.org/interactive) was referred to predict the spatial structure of amino acid peptide chain from 206th aa to 725th aa in *k13* gene, which was obtained from the translation of the PCR amplification product. The reference model was 4zgc.1.A. The identity of the model approaches to 100%, sequence similarity, coverage and GMQE are closer to 1, and the smaller value of QMEAN, jointly indicates higher quality of the spatial prediction of the peptide chain. The spatial structure prediction graph was edited and modified by processing PDB format data using PyMOL 2.2.0 software.

## Results

### PCR amplification of *k13* gene

**Fig. 1:**
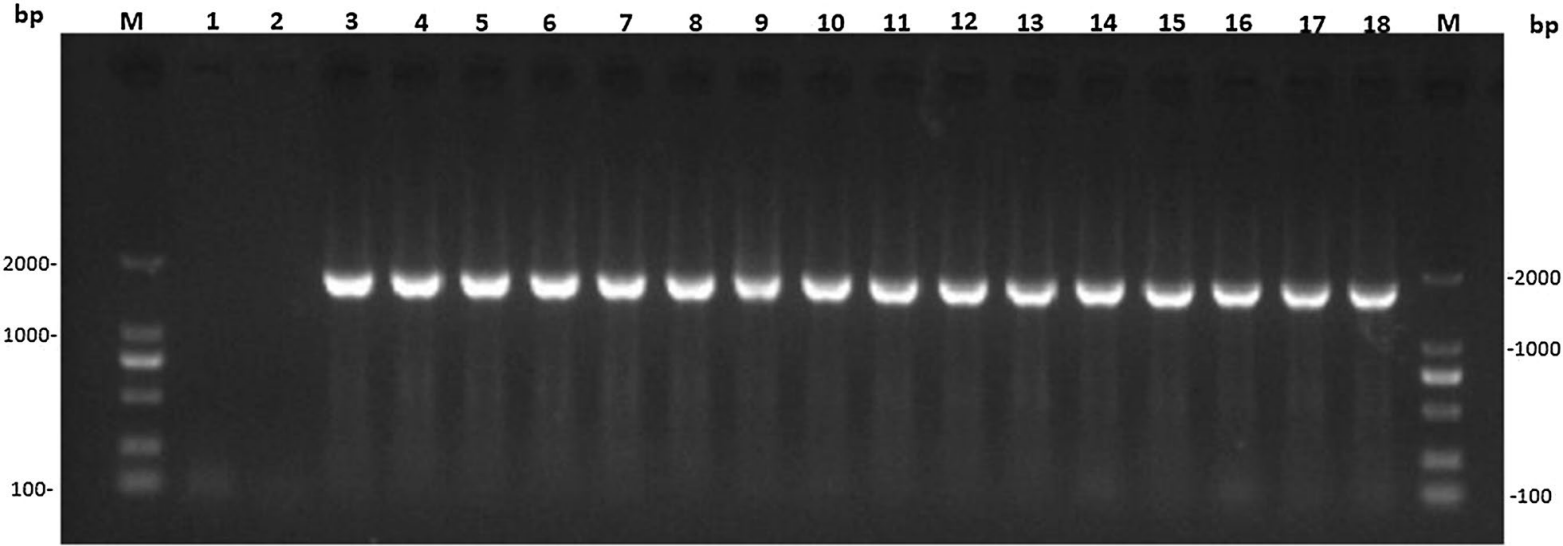
PCR amplification of the coding region (from 206th aa to 725th aa) in *k13* gene from blood samples of ovale malaria patients. Lane 1: Blank control in first-round PCR amplification. Lane 2: Blank control in second-round PCR amplification. Lane 3: Positive control of PCR amplification. Lane 4–18: *k13* gene fragment amplification product of sample. M stands for DNA maker

Eighteen blood samples from malaria cases mono-infected with *P. ovale* were collected and processed, and the genomic DNA of the blood samples was subjected to nested PCR amplification form 206th aa to 725th aa in the coding region of *k13* gene. In total, 15 samples of electrophoretic amplification products of second-round PCR were obtained in a length of 1732 bp (Fig. 1). The target band showed a positive amplification rate of 83.3% (15/18). The other three samples (3/18) were not included in the bioinformatics analysis because of substandard quality of sequencing.

Among fifteen samples included, all of which were initially identified as *P. vivax* infection at county-level laboratory in Yunnan province. Then, YNRL confirmed them as *P. ovale* infection (Additional file 1). Of the 15 cases, 10 cases were infected in African countries, such as Republic of the Congo, Gabon, Guinea, Nigeria, Cameroon, Uganda and Ghana, and 5 cases were infected in Myanmar (Table 1). All these cases were male, aged between 27 and 45 years old.

**Table 1:** Information of 15 *ovale* malaria cases with their *Plasmodium* species distinguished by *k13* gene dimorphism

Infection source^a^	*P. vivax*^b^	*P. ovale*^c^	Years						*P. ovale spp.*		
			2013	2014	2015	2016	2017	2018	*curtisi*	*wallikeri*	mutation
Total	15	15	2	3	2	1	2	5	11	3	1
Myanmar	5	5	2	3	0	0	0	0	5	0	0
Congo	2	2	0	0	1	1	0	0	1	0	1
Gabon	1	1	0	0	0	0	0	1	0	1	0
Guinea	2	2	0	0	0	0	1	1	2	0	0
Nigeria	1	1	0	0	1	0	0	0	1	0	0
Cameroon	2	2	0	0	0	0	0	2	1	1	0
Uganda	1	1	0	0	0	0	1	0	0	1	0
Ghana	1	1	0	0	0	0	0	1	1	0	0

^a^Identified by epidemiological investigation; ^b^Species initially identified by county-level laboratories in Yunnan Province; ^c^Species confirmed by YNRL in Yunnan Province

### Polymorphism analysis of coding DNA region in *k13* gene

The PCR sequencing results of the 15 samples were aligned to obtain 15 CDSs belonging to the domain from 224th aa to 725th aa in *k13* gene (GenBank accession numbers: MT430952-MT430966). The value of Ka / Ks was 3.784, and there were three different polymorphic haplotypes (Hap_01–Hap_03) in these sequences. The nucleic acid diversity index (π) was 0.0095, and the expected heterozygosity (He) was 0.4250.

**Fig. 2:**
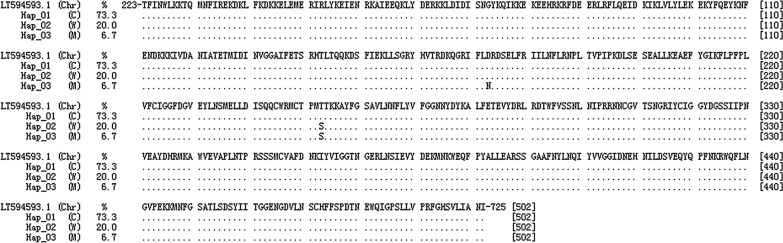
Alignment of the amino acid chains encoded by *k13* gene of *P. ovale.* (1) Hap_01, Hap_02 and Hap_03 indicate the haplotype of the samples. (2) Chr: The reference sequence from chromosome. (3) C: *curtisi* subtype. (4) W: *wallikeri* subtype. (5) M: Mutation type

Hap_01 haplotype was *curtisi* subtype, which accounted for 73.3% (11/15). Among them, 5 isolates were from Myanmar, and 6 were from Africa. Hap_02 haplotype was *wallikeri* subtype sequences, which accounted for 20.0% (3/15), and were Africa-imported isolates (Table 1). Compared with *curtisi* subtype sequences, *wallikeri* subtype sequences showed base substitutions at 38 loci, such as c.711, and c.1086. (Table 2). The substitutions of the 3rd and 1st bases belonging to triplet codon accounted for 92.1% (35/38) and 7.9% (3/38) respectively. At c.1428 locus, the A > ***T*** conversion in the 1^st^ base led to 476 codon (ACA > ***T***CA) forming nonsynonymous mutation, which showed a T476S variation at 476th aa (Fig. 2). Hap_03 haplotype was a mutant type, which accounted for 6.7% (1/15). In comparison with the sequences of *wallikeri* subtype, it had only a base substitution of G > ***A*** at c.1186 loci, resulting GAT > ***A***AT nonsynonymous mutations in 396 codon and forming D396N variation at 396th aa (Fig. 2).

**Table 2:** Polymorphism comparison of *P. ovale curtisi* and *P. ovale wallikeri* in the propeller domain of *k13* Genes from 224th aa to 725th aa

SN^a^	Loci	BS^b^	Codon change	Variation	SN	Loci	BS	Codon change	Variation
1	c.711	T > A	ATT > ATA	I237I	20	c.1557	T > C	TTA > CTA	L523L
2	c.1086	A > T	ACA > ACT	T362T	21	c.1578	A > T	CCA > CCT	P526P
3	c.1116	C > T	GAC > GAT	D372D	22	c.1707	G > T	CCG > CCT	P569P
4	c.1173	T > A	GGT > GGA	G391G	22	c.1731	C > T	TCC > TCT	S577S
5	c.1186	G > ***A***	GAT > ***A***AT	D396N	24	c.1740	A > C	GTA > GTC	V580V
6	c.1204	T > C	TTA > CTA	L402L	25	c.1758	A > T	ATA > ATT	T586T
7	c.1263	G > A	TTG > TTA	L421L	26	c.1896	A > T	TCA > TCT	S623S
8	c.1281	G > A	TTG > TTA	L427L	27	c.1908	T > G	GTT > GTG	V636V
9	c.1296	G > A	GAG > GAA	K432K	28	c.1935	C > T	ATC > ATT	I645I
10	c.1305	C > T	GGC > GGT	G435G	29	c.1941	T > C	GAT > GAC	D647D
11	c.1365	T > C	TAT > TAC	Y455Y	30	c.1947	A > G	GTA > GTG	V649V
12	c.1386	G > A	TTG > TTA	L462L	31	c.1959	A > G	CAA > CAG	Q653Q
13	c.1389	T > C	GAT > GAC	D463D	32	c.1992	G > A	GGG > GGA	G664G
14	c.1422	A > T	CCA > CCT	P474P	33	c.2001	A > G	GAA > GAG	E667E
15	c.1428	A > ***T***	ACA > ***T***CA	T476S	34	c.2058	A > G	GGA > GGG	G686G
16	c.1440	A > T	GCA > GCT	A480A	35	c.2073	A > C	GTA > GTC	V691V
17	c.1455	A > T	GCA > GCT	A485A	36	c.2082	T > C	TCT > TCC	S694S
18	c.1548	C > A	ACC > ACA	T516T	37	c.2112	A > G	GAA > GAG	E704E
19	c.1554	T > C	TTT > TTC	F518F	38	c.2118	A > G	CAA > CAG	Q706Q

^a^Sequence number; ^b^Base substitution

### Spatial prediction of the peptide chain of *k13* gene

The spatial prediction diagram was constructed based on the amino acid peptides translated from the CDSs from 224th aa to 725th aa in *k13* gene. The sequences of *curtisi* subtype and *wallikeri* subtype can only form the monomeric model, while the sequences of both c.1186 and c.1428 double-site nonsynonymous samples can form the dimeric model. The amino acid peptide chains in the model ranged from 126th aa to 502th aa, corresponding to 249th aa to 725th aa in *k13* gene. Moreover, the 125 amino acids at the N-terminus cannot be modelled. Therefore, the sequence similarity and coverage of the sample sequence and origin for the “reference model (4zgc.1.A)” were merely 0.61 and 0.77–0.79, respectively. However, the GMQE values of the four models were close to each other, ranging from 0.73 to 0.74. The absolute values of QMEAN were all less than 0.06 (Table 3). These data collectively indicate that the quality of the spatial model of various peptide chains is similar and sound.

**Table 3:** Model parameters of predicted spatial structure of* k13* kelch protein of *P. ovale*

Amino acid sequence	Oligo state	Amino acids range of model	GMQE	QMEAN	Identity (%)	Sequence similarity	Coverage
Referent model			(4zgc.1.A)				
LT594593.1	Monomer	126–502	0.73	− 0.06	97.69	0.61	0.79
Hap_01	Monomer	126–502	0.74	− 0.01	97.43	0.61	0.77
Hap_02	Monomer	126–502	0.73	− 0.06	97.69	0.61	0.77
Hap_03	Homodimer	126–502	0.74	0.03	97.13	0.61	0.77

**Fig. 3:**
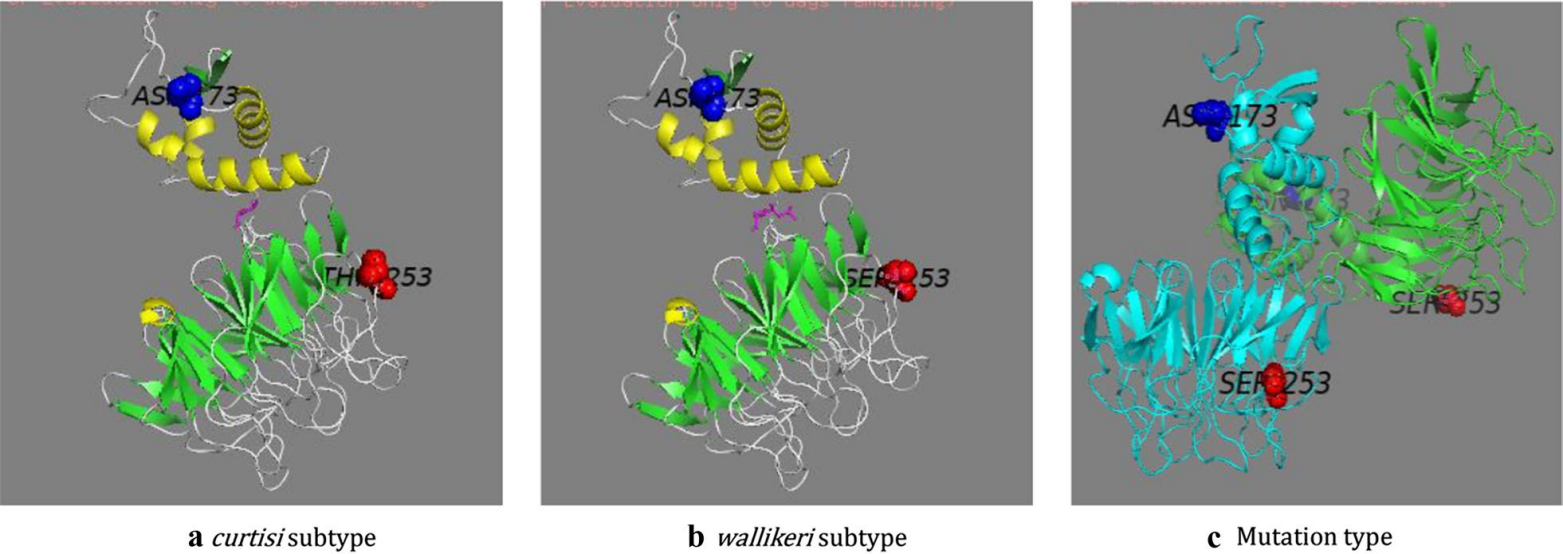
Spatial prediction diagram of the amino acid peptide chains of *k13* gene from 224th aa to 725th aa

The monomeric spatial models of both *curtisi* subtype and *wallikeri* subtype peptide chains show that with 216th aa to 217th aa (corresponding to 438th aa to 439th aa in k13 gene) serving as the separation point, the nearer the N-terminus exhibited an α-helix structure and the nearer the C-terminus displayed a β- helix structure from 224th aa to 725th aa peptide chains. The 476th aa was located on the surface of β-sheet structure, yet the variation of T476S does not affect the formation of the spatial structure of the peptide chain (Fig. 3a, b). The 396th aa was located inside the α-helical structure, and its variation to D396N induced the formation of dimeric spatial structure of the peptide ranging from 224th aa to 725th aa in *k13* gene (Fig. 3c).

## Discussion

The *k13* gene of *P. ovale* is in the 404824-407001 rt region of chromosome 12, with a coding region in full length of 2178 bp. Its encoded kelch protein has a skeletal region near the N-terminus, and a propeller domain near the C-terminus consisting of about 290 amino acids from 440th aa-725th aa [26]. Studies have shown that amino acid substitutions in the propeller domain of the kelch protein in *P. falciparum* are genetically related to artemisinin resistance [25, 27]. Moreover, there are very few bases with more than two substitution loci in the entire coding region, which demonstrates [25, 28, 29] high conservation. Therefore, *k13* gene can be used as a stable molecular marker to predict the artemisinin resistance in *P. falciparum* [30–32].

In this study, the polymorphism of the entire propeller domain and a fraction of the upstream skeletal domain in *k13* gene of the *P. ovale* isolates imported into Yunnan Province from Myanmar and some African countries were analysed. Of the 15 CDS sequences analysed, base substitutions were found at 38 loci, such as c.711–c.2118 (Table 2), showed the inter-type dimorphism of *curtisi* subtype and *wallikeri* subtype, as well as the complete intra-type monomorphism (Fig. 2). The finding of such stable monomorphism and dimorphism characteristics at each locus is consistent with the results of polymorphism analysis conducted by Sutherland et al*.* [12], Fuehrer et al. [33], Chavatte et al*.* [7] on reticulocyte-binding protein 2 gene (*rbp2*), and glyceraldehyde-3-phosphatase (*g3p*) gene. All the above-mentioned research found the dimorphism of different genes in *P. ovale,* such as at 22 loci in *rbp2* gene with approximately a 793 bp fragment and at 19 loci in *g3p* gene with a 662 bp fragment between *curtisi* subtype and *wallikeri* subtype sequences. Moreover, the loci showed highly monomorphic within *curtisi* subtype and *wallikeri* subtype sequences. While the *P. ovale* tryptophan-rich antigen gene (*Potra*) in *wallikeri* subtype had a 54-bp deletion compared to it in the *curtisi* subtype [12]. Fuehrer et al*.* [33] also found there were different dominant short peptide chain repeat in circumsporozoite surface protein gene (*csp*) between *curtisi* subtype and *wallikeri* subtype. For the *csp* gene of *wallikeri* subtype, the "DPPAPVPQG" short peptide chain was more frequent, while for *curtisi* subtype was the "NPPAPQGEG" short peptide chain. It seems that the polymorphism of *csp* gene could be used to establish the genotyping method for distinguishing two subtypes, but Fueher et al*.* [33] believed it was only applicable to determination of *P. ovale* evolutionary relationships. In the current research, the authors discovered the dimorphism at 38 base loci in *k13* gene propeller domain existed between two subtypes and the CDSs of *curtisi* subtype (n = 11) and *wallikeri* subtype (n = 4) could be separated in the Neighbour-Joining evolutionary tree. However, on account of the failure to find the predisposing structural features like *csp* gene in CDSs or peptide chain of *k13* gene propeller domain. Therefore, it is difficult to establish a suitable discriminating method between *curtisi* subtype and *wallikeri* subtype based on the polymorphism of *k13* gene, propeller domain, and further analysis on the backbone domain of *k13* gene might be helpful to find more evidence.

Consequently, these findings about *k13* gene in this article only emphasize that *k13* gene polymorphism in *P. ovale* is like the differentiation of other members in the genome, resulting in the distinction between *curtisi* subtype and *wallikeri* subtype. However, it is noted that the degree of *k13* gene differentiation is weaker than circumsporozoite protein / thromspondin-related anonymous protein (*ctrp*), circumsporozoite protein (*csp*) and merozoite surface protein 1 gene (*msp1*), which were reported by Saralamba et al*.* [34]. The Pi value of these three genes was predicted to be between 0.12 and 0.11, which is greater than 0.0095 in this study.

Evidence indicated that *P. ovale* originated from Southeast Asian countries is mostly *curtisi* subtype, while Africa showed a sympatric distribution of *P. o. curtisi* and *P. o. wallikeri* [7, 12, 35–37], and the mutation type is mainly restrained in Western Africa [20]. In this study, the distribution pattern of similar *P. ovale* subspecies was almost restored. The sequences of *k13* propeller domain in 5 Myanmar isolates were all identified as *curtisi* subtype, while the 10 African isolates included six *curtisi* subtype, three *wallikeri* subtype and one mutation type (Table 1). This result serves as a constant reminder that the population structure of *P. ovale* isolates imported into Yunnan province maybe are more complicated than those of the original population [34, 38]. Therefore, greater discretion and accuracy are needed in the diagnosis and antimalarial treatment of these *P. ovale* infections. The current study is the first to ascertain that the infected isolates in malaria cases officially reported in Yunnan Province include the 2 sub-species of *P. ovale curtisi* and *wallikeri* and further providing a favourable basis for the control of ovale malaria epidemic in Myanmar [39]. In addition, although amino acid substitution variation in the skeleton region of kelch protein was detected in only one sample, the same amino acid variation has also detected and demonstrated by Jin’s study on the samples from Hangzhou city, China (personal communication), which increase credence of the result. However, double DNA sequencing process could further help rule out sequencing errors.

Although this study was not dedicated to exploring the genetic correlation between *k13* gene mutations and artemisinin resistance in *P. ovale*, the spatial structure prediction on the peptide chain near the C-terminus from 224th aa to 725th aa in *k13* gene found that *curtisi* subtype peptide chains and *wallikeri* subtype peptide chains share almost analogous monomeric crystal structures (Fig. 3a, b). Moreover, with 1 amino acid variation in the skeleton region, yet the homology model has dramatically changed into a dimeric structure (Fig. 3c). The finding is completely different from that of Choowongkomon et al*.* [37] in terms of the spatial structure prediction of dihydrofolate reductase (*dhfr*) gene in *P. ovale*. Their results showed the identities of *dhfr* peptide chain in *P. ovale* were merely 67.4, 64.7 and 75.4% in comparison with *P. vivax*, *P. falciparum*, and *P. malariae,* respectively. However, the crystal structures of the four *dhfr* peptide chains are similar regarding subunit composition and the tendency of overall folding. All display monomeric and α-helix structure, which are folded on the surface of the homology model [36]. This pattern might be related to the different proportions and intensities of α-helix and β-helix structures in the two peptide chains of *k13* gene and *dhfr* gene. In the current study, β-helix structures accounted for 75.1% (377 aa / 505 aa) in the *k13* peptide chain, and were mainly located in the C-terminus of the peptide chain to fold into a "propeller" shape. In addition, Bayih et al*.* [40] had proposed the substitution from basic-to-aliphatic residue at the kelch 13 propeller domain, especially β-helix structures region, may impact the protein function. However, further studies should be carried out to investigate whether the predicted structural change in skeletal region of the kelch protein in *P. ovale*, just like the mutation of the propeller domain, is related to the artemisinin-resistant phenotype [25, 27].

In this study, the understanding that there are numerous dimorphisms in the genome of *P. o. curtisi* and *P. o. wallikeri* was broadened. By using the multi-loci dimorphism of the *k13* gene, it might be possible to establish a stable and accurate genotyping method for distinguishing different subtypes of *P. ovale*. Nevertheless, this study is not without limitations. Firstly, given the difficulty to accurately calculate the parasitaemia of *P. ovale* in some blood slides, it is impracticable to explore the correlation between the density of the parasites and the copy number of *k13* gene. Secondly, the polymorphic analysis of the full sequence of the *k13* gene has not yet been performed, and the incomplete identification of the dimeric loci in the skeleton region of kelch protein and the DNA sequence of *P. o*. *curtisi* and *P. o. wallikeri* might not be conducive to assess of the degree of *k13* gene differentiation accurately. Thirdly, this protocol is based on nested PCR and DNA sequencing, which is labour- and cost-intensive due to the second PCR reaction, and also increase risk of contamination due to PCR product transfer from the initial reaction to the second, while one-step real time PCR assay to discriminate *P. ovale* subspecies using specific primers and hydrolysis probes targeting *rbp2* gene has been reported and applied in West Kenya [41]. Lastly, given 16.7% (3/18) of the samples fail to be detected by this protocol, low parasitaemia of these samples (not counted due to poor quality of slides) might be one cause, or potentially due to multiple reference sequences were not included as template during the primer design stage, which could limit the sensitivity of the experiment. Local wild type sequences could be used as reference to design the primers to increase the sensitivity of this protocol.

## Conclusion

The propeller domain of *k13* gene in the *P. ovale* isolates imported into Yunnan from Myanmar and Africa was largely differentiated, yet most of the base substitutions still belong to synonymous mutation. All the sequences of Myanmar-imported isolates were *P. o. curtisi*, while the sequences of Africa-imported isolates showed the sympatric distribution of *P. o. curtisi* and *P. o. wallikeri* subtypes, as well as mutation types. The CDS sequence with double base nonsynonymous substitution has a spatial structure to encode dimeric peptide chain, which is completely different from the monomeric spatial structure of peptide chains encoded by *P. o. curtisi* and *P. o. wallikeri*. The polymorphism analysis of *k13* gene sequence was used for the first time to confirm that all the Myanmar-imported isolates were *P. ovale curtisi* subtype, which could be helpful for the accurate diagnosis and clinical intervention of ovale malaria in the country.

## Supplementary information

**Additional file 1.** Malaria case confirmation by Yunnan Provincial Reference Laboratory.

## Data Availability

Not applicable.

## Abbreviations

YNRL: Yunnan Province Reference Laboratory
DBS: Dried blood spots
NSS: Nonsynonymous substitution
SS: Synonymous substitution
BS: Base substitution
PCR: Polymerase chain reaction
CDS: Coding DNA sequence
18S rRNA: 18S (small subunit) ribosomal RNA gene
*rbp2*: Reticulocyte binding protein 2
*g3p,*: Glyceraldehyde-3-phosphatase gene
*ctrp*: Circumsporozoite protein/ thrombospondin-related anonymous protein
*csp*: Circumsporozoite surface protein
*msp1*: Merozoite surface protein 1
*dhfr-ts*: Dihydrofolate reductase thymidylate synthase gene
*Potra*: Tryptophan rich antigen gene
