# Supplementary material for: Polymorphism analysis of propeller domain of k13 gene in Plasmodium ovale curtisi and Plasmodium ovale wallikeri isolates original infection from Myanmar and Africa in Yunnan Province, China
Source: Malar J. 2020 Jul 13;19:246. doi: 10.1186/s12936-020-03317-2 (PMC7359257; doi:10.1186/s12936-020-03317-2)
Supplement: Supplementary file 1 — Additional file 1. Malaria case confirmation by Yunnan Provincial Reference Laboratory. [file 12936_2020_3317_MOESM1_ESM.pdf]

Supplement 1

Malaria case confirmation by Yunnan Provincial Reference Laboratory

All the confirmed *P. ovale* cases in Yunnan Province have been misidentified as *P. vivax* by the routine morphological examination performed at the county-level laboratories. The morphological and genetic data for case confirmation at YNRL of 15 malaria cases were as follows.

Morphological examination results

1. rld01m (*P. ovale*，thin blood smear, ×1000)

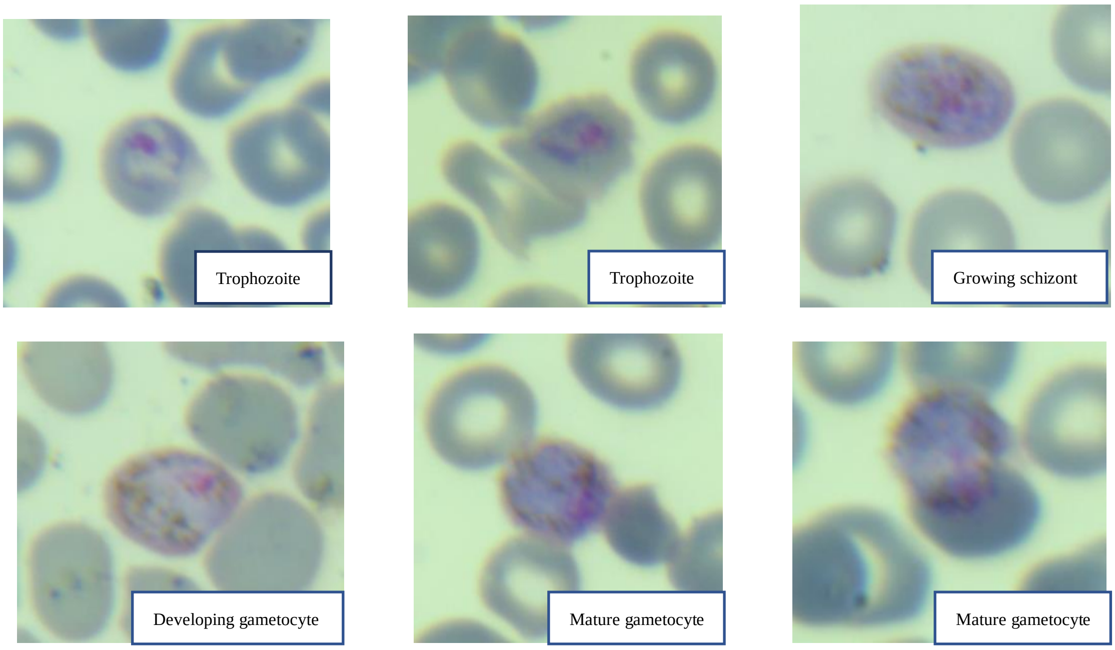

2. tcb02m (*P. ovale*，thin blood smear, ×1000)

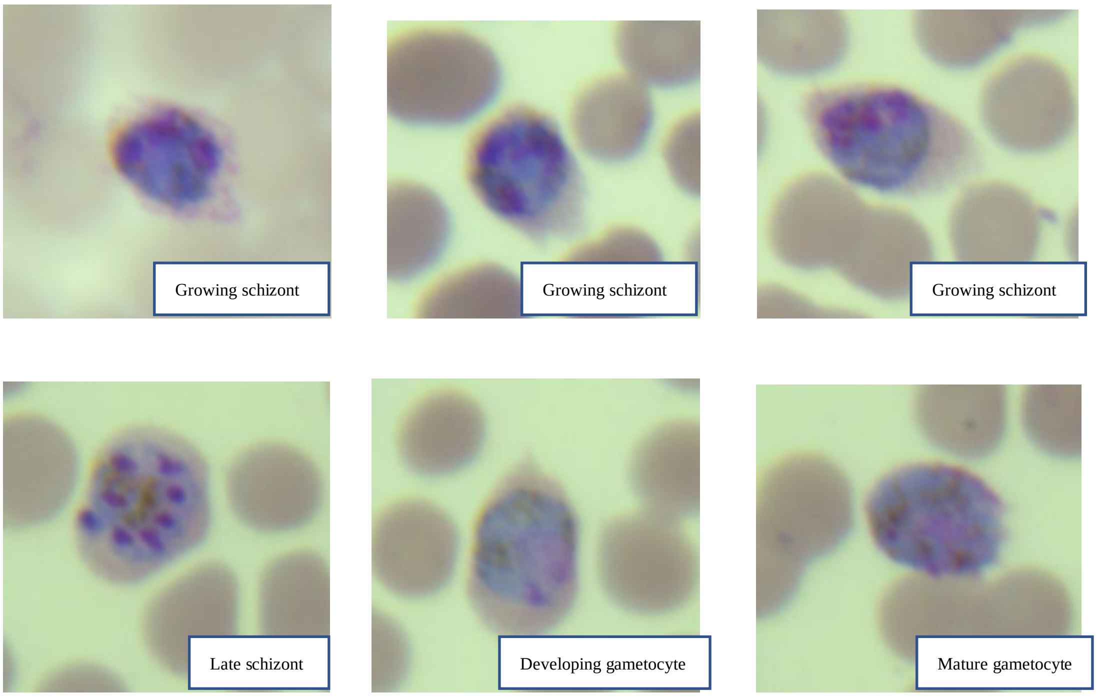

3. lcd03m (*P. ovale*, thin blood smear, ×1000)

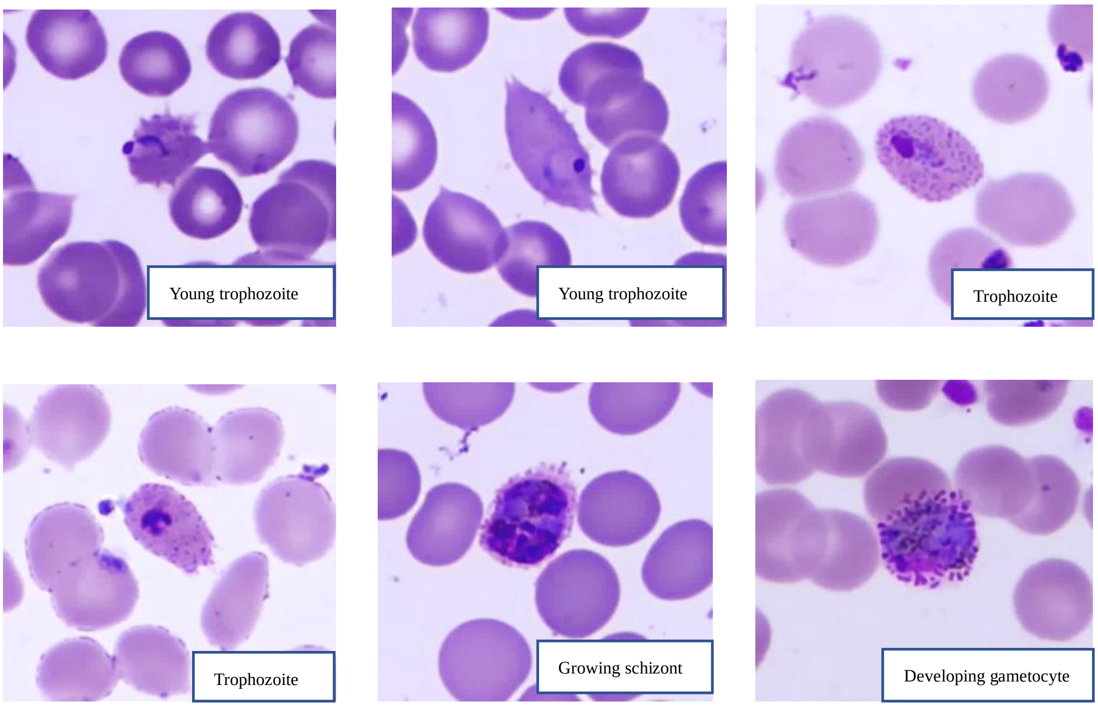

4. ltcb04m (*P. ovale*, thin blood smear, ×1000)

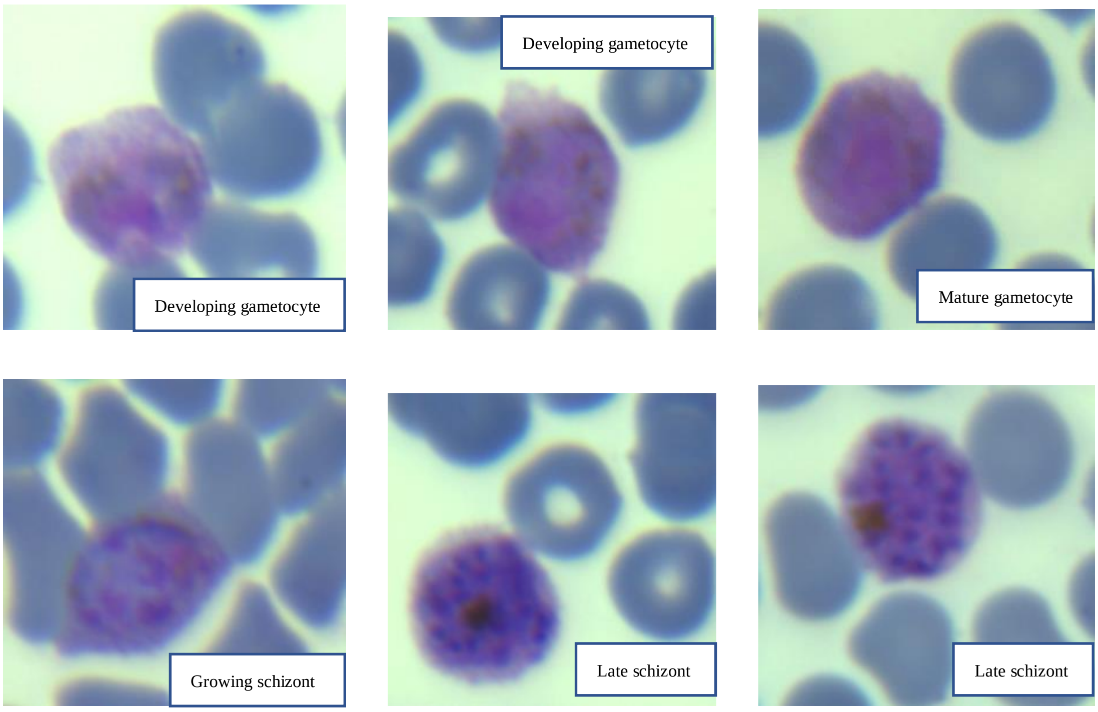

5. gdk05m (*P. ovale*， thin blood smear, ×1000)

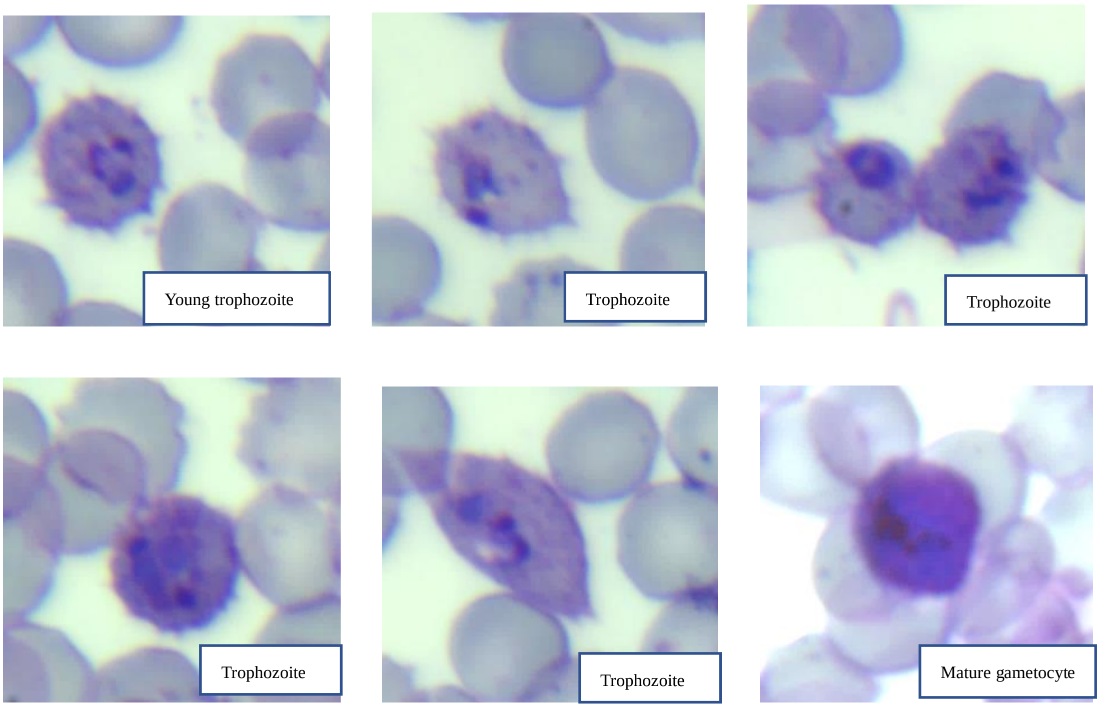

6. kgk06a (*P. ovale*， thin blood smear, ×1000)

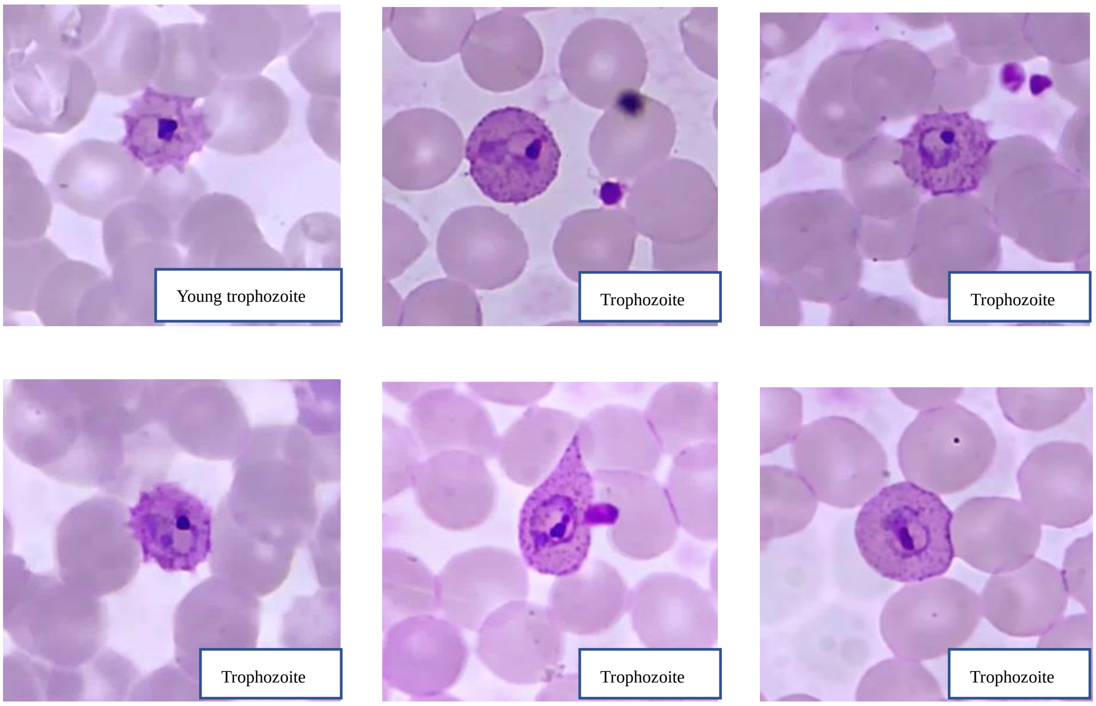

7. gsk07a (*P. ovale*，thin blood smear, ×1000)

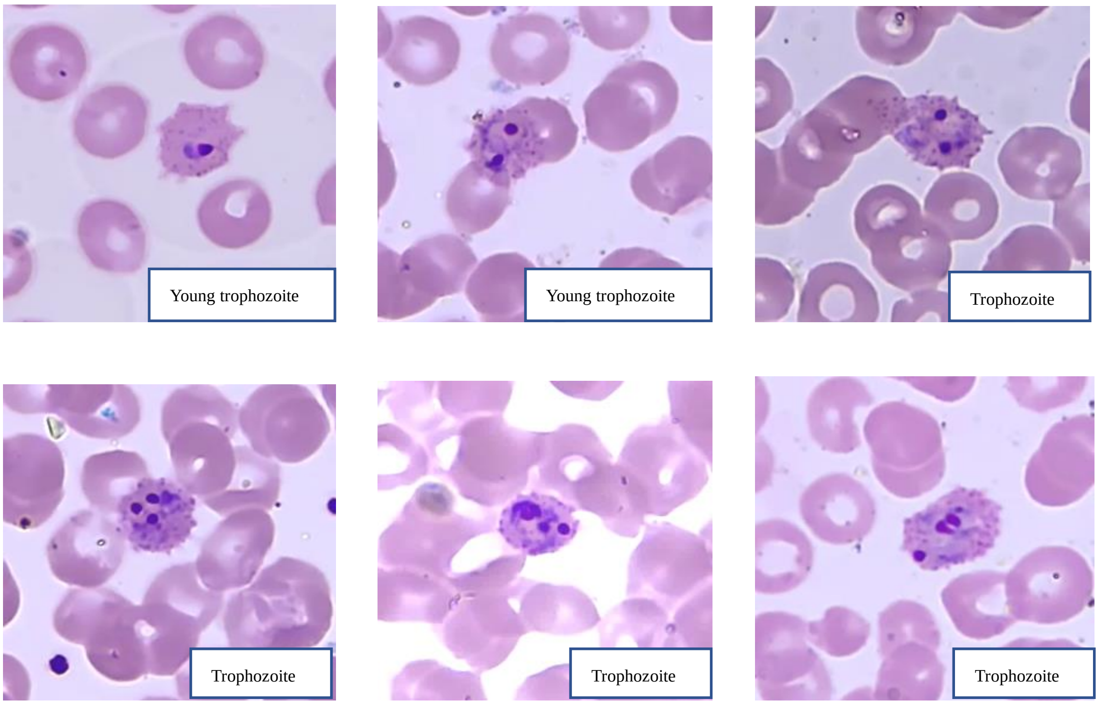

8. dld08a (*P. ovale*，thin blood smear, ×1000)

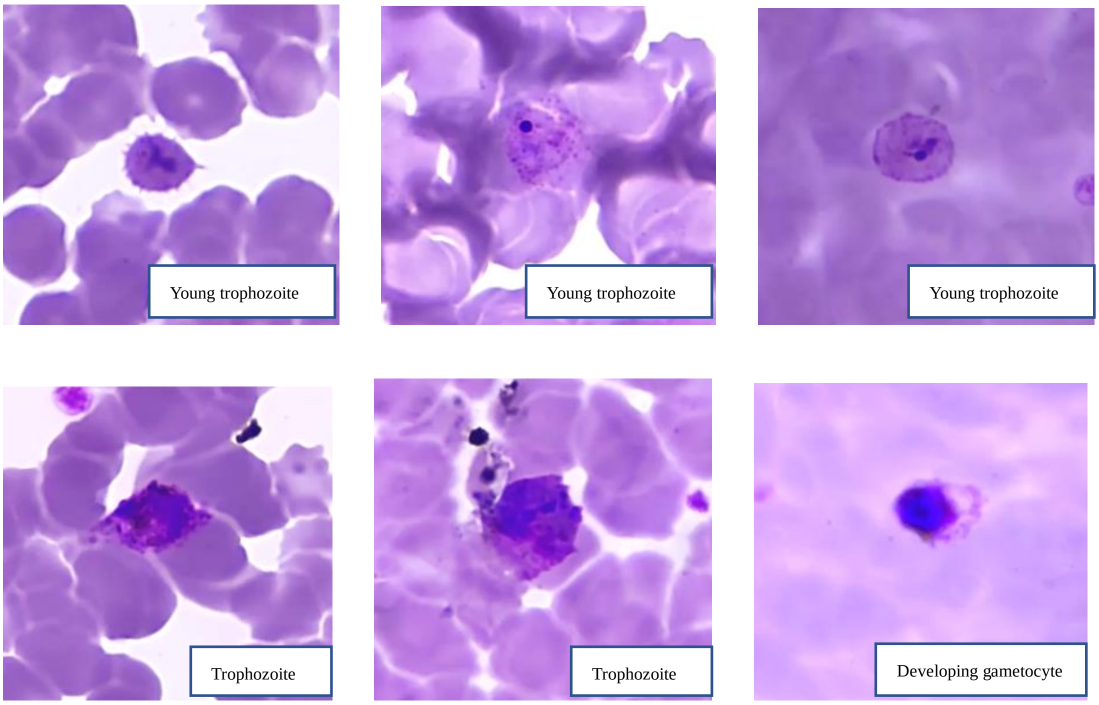

9. jsh09a (*P. ovale*， thin blood smear, ×1000)

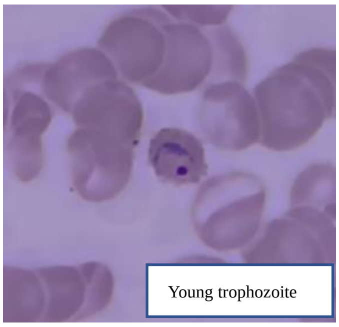
Young trophozoite

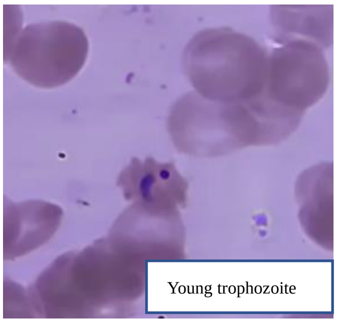
Young trophozoite

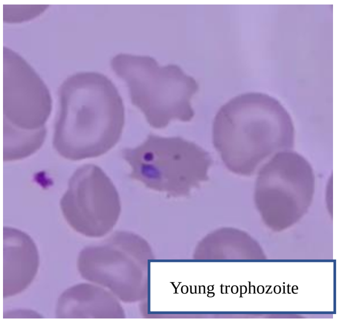
Young trophozoite

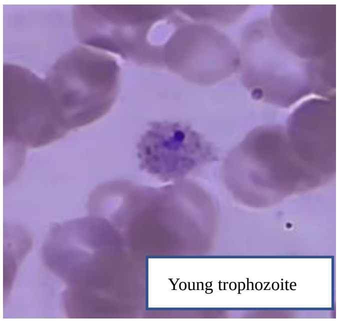
Young trophozoite

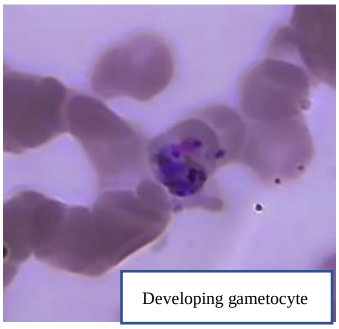
Developing gametocyte

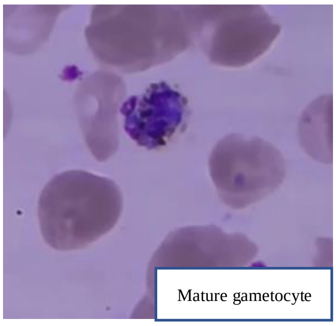
Mature gametocyte

10. ztz10a (*P. ovale*， thin blood smear, ×1000)

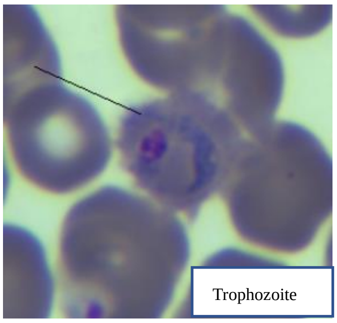
Trophozoite

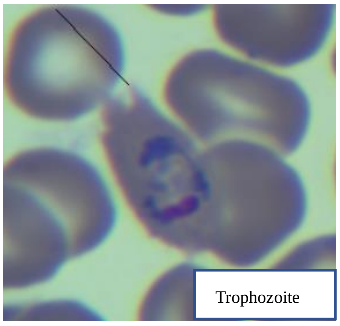
Trophozoite

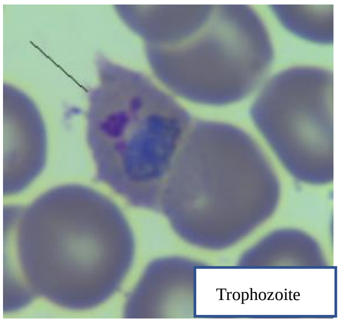
Trophozoite

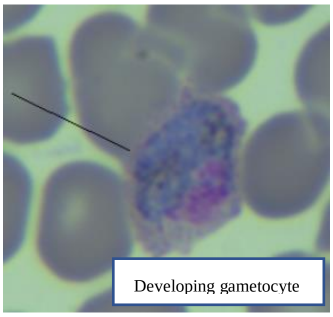
Developing gametocyte

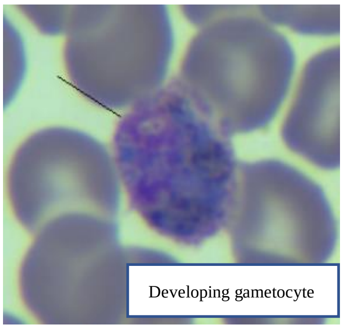
Developing gametocyte

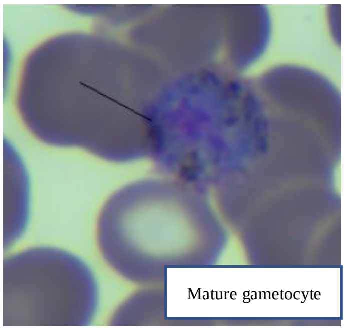
Mature gametocyte

11. ldz11a (*P. ovale*，thin blood smear, ×1000)

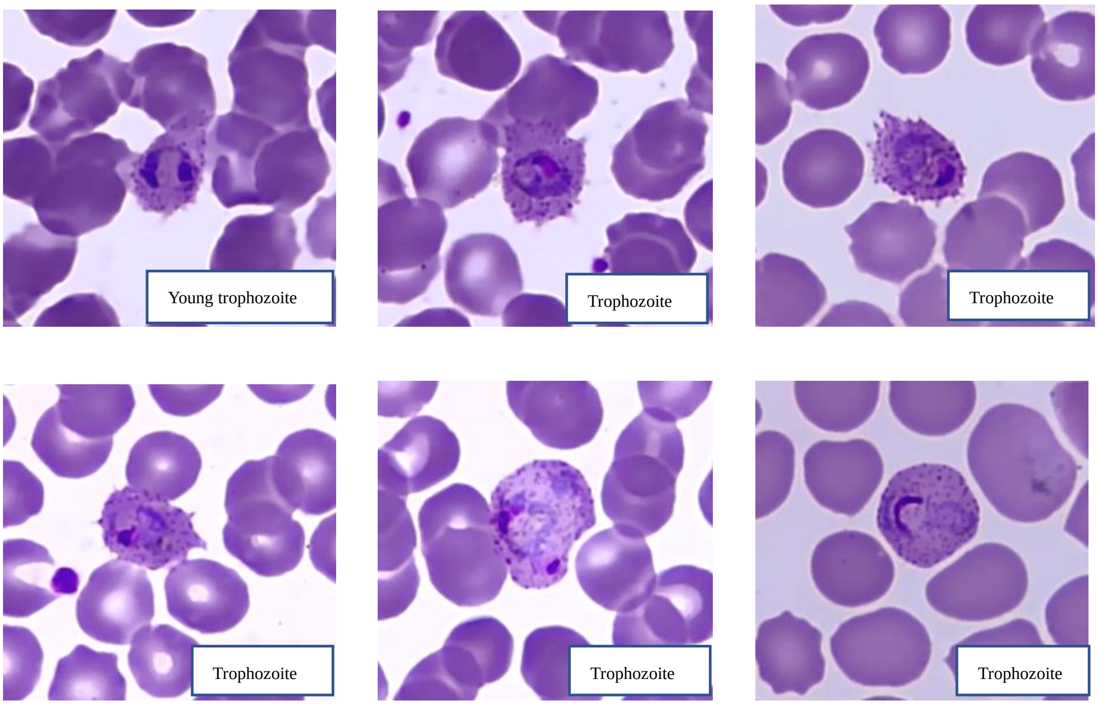

12. esy12a (*P. ovale*，thin blood smear, ×1000)

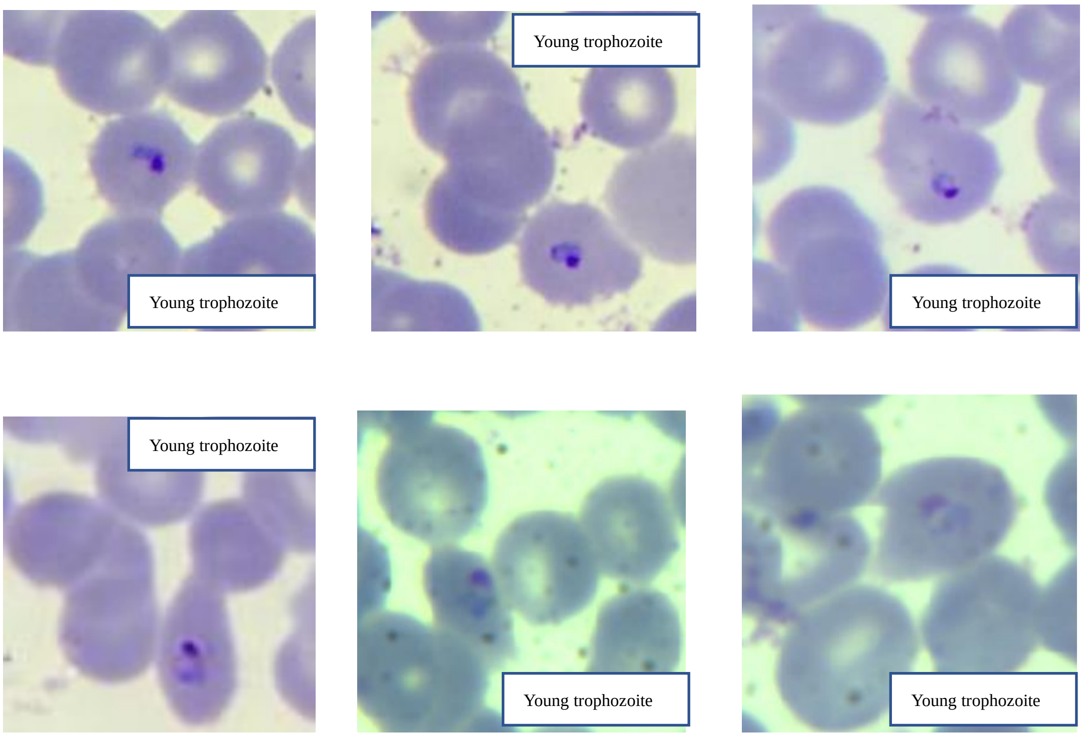

13. jcy13a (*P. ovale*，thin blood smear, ×1000)

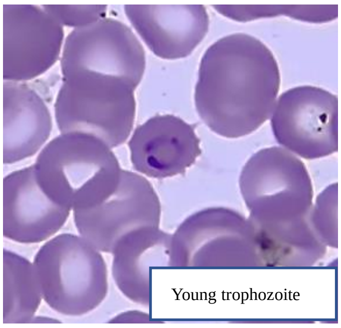
Young trophozoite

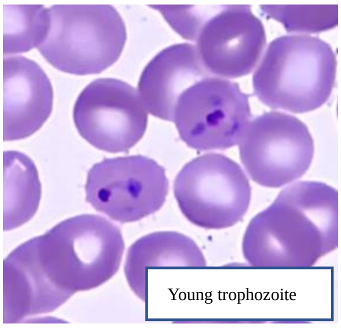
Young trophozoite

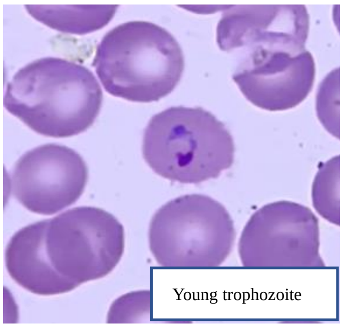
Young trophozoite

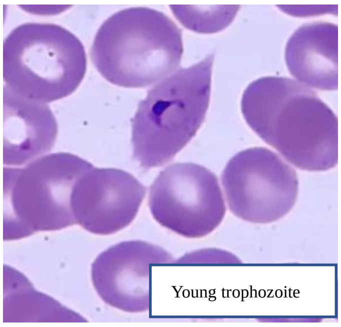
Young trophozoite

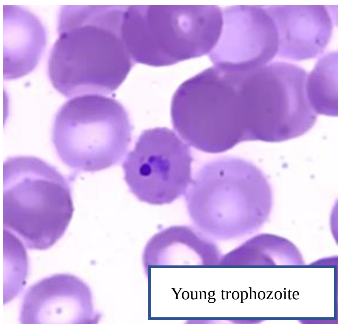
Young trophozoite

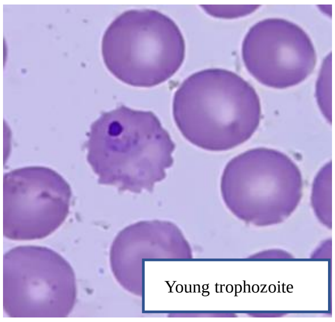
Young trophozoite

14. dld15a (*P. ovale*，thin blood smear, ×1000)

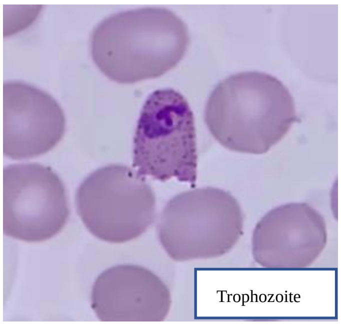
Trophozoite

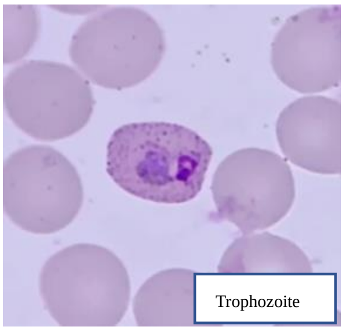
Trophozoite

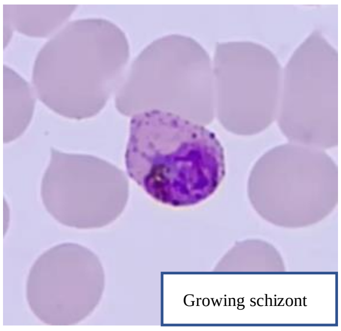
Growing schizont

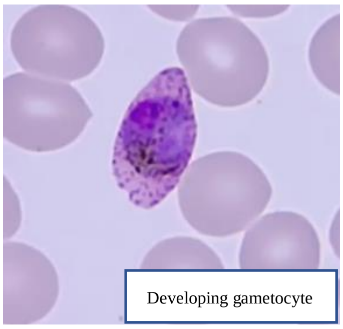
Developing gametocyte

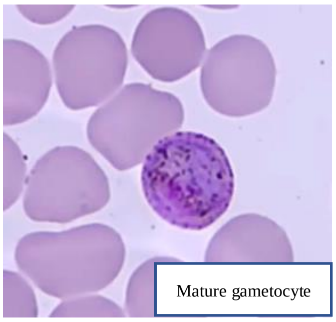
Mature gametocyte

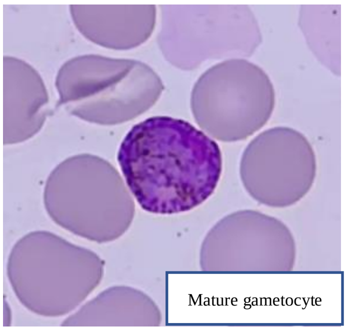
Mature gametocyte

15. xwq17a (*P. ovale*，thin blood smear, ×1000)

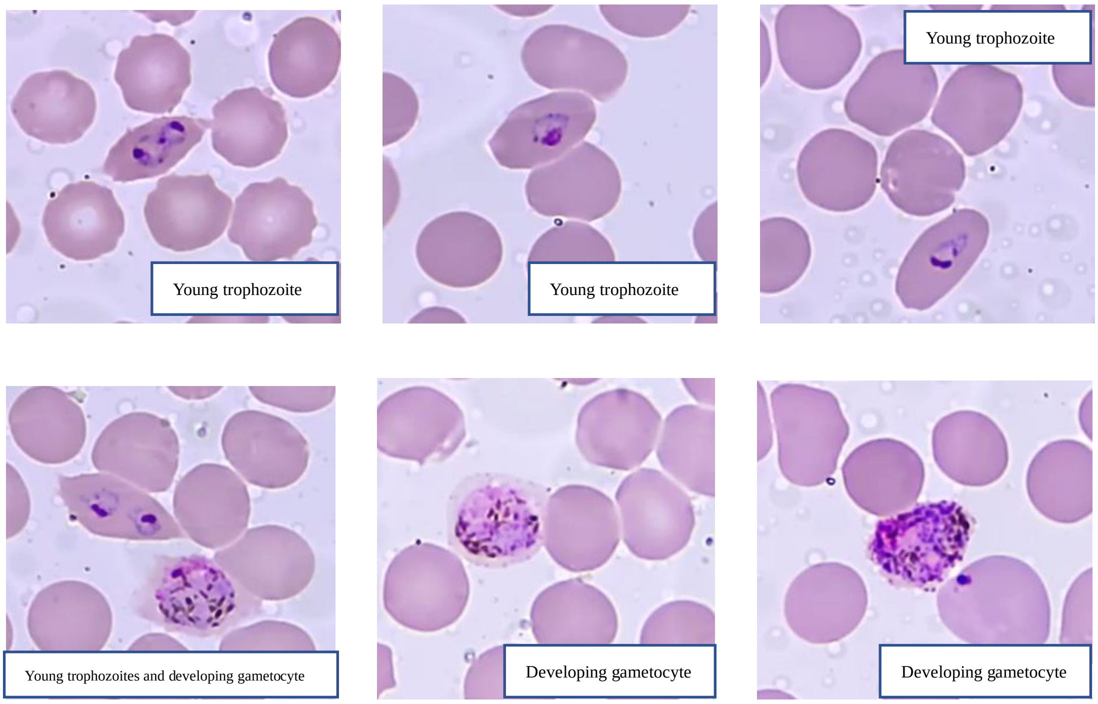

PCR results

The target gene was 18S （small subunit） ribosomal RNA gene (18S *rRNA*). The upstream, downstream primers of first-round and second-round PCR reaction were 5'-CCTGTTGTTGCCTTAAACTTC-3' and 5'-TTAAAATTGTTGCAGTTAAAACG-3', 5'-ATCTCTTTTGCTA TTTTTTAGTATTGGAGA-3' and 5'-GGAAAGGACACATTAATTGTATCCTAGTG-3', respectively. The expected DNA fragments of the amplified product in second-round PCR was 800 bp.

The following electropherogram showed that the amplifications of *P. ovale* 18S rRNA gene in the 15 isolates (rld01m, tcb02m, lcd03m, tcb04m, gdk05m, kgk06a, gsk07a, dld08a, jsh09a, ztz10a, ldz11a, esy12a, jcy13a, dld15a, xwq17a) was positive.

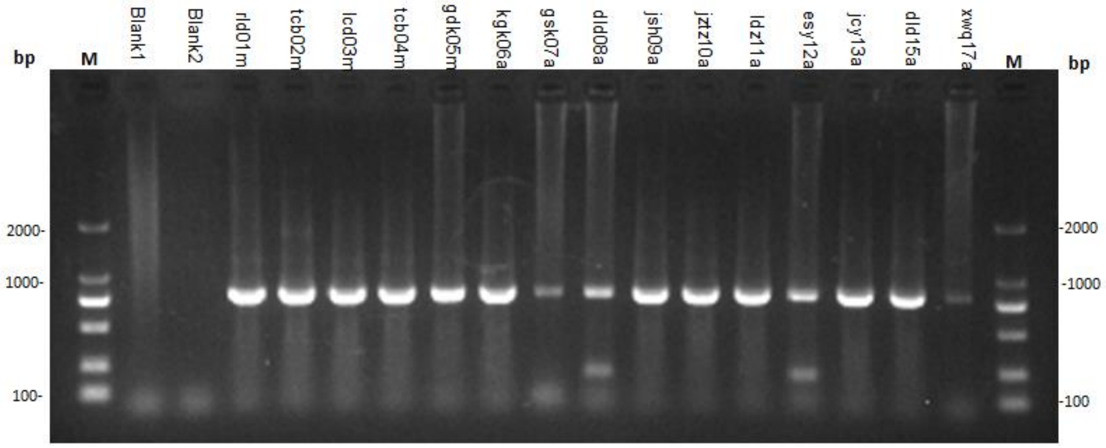

Species further confirmed by Yunnan Provincial Reference Laboratory (Table 1)

Table 1 The records of case confirmation for 15 *ovale* malaria cases

Samples	Infection source [a]	Initial diagnosis		Further diagnosis [b]		
		Species	County-level Lab.	Species by microscope examination	Parasitemia (parasites/μl)	Species by genetic detection
rld01m	Myanmar	*P. vivax*	Ruili	*P. ovale*	77	*P. ovale*
tcb02m	Myanmar	*P. vivax*	Tengchong	*P. ovale*	44730	*P. ovale*
lcd03m	Myanmar	*P. vivax*	Longchuan	*P. ovale*	475	*P. ovale*
tcb04m	Myanmar	*P. vivax*	Tengchong	*P. ovale*	96	*P. ovale*
gdk05m	Myanmar	*P. vivax*	Guandu	*P. ovale*	16898	*P. ovale*
kgk06a	Congo	*P. vivax*	Guandu	*P. ovale*	10082	*P. ovale*
gsk07a	Gabon	*P. vivax*	Guandu	*P. ovale*	6322	*P. ovale*
dld08a	Congo	*P. vivax*	Dali	*P. ovale*	14717	*P. ovale*
jsh09a	Guinea	*P. vivax*	Jianshui	*P. ovale*	38	*P. ovale*
ztz10a	Nigeria	*P. vivax*	Zhaotong	*P. ovale*	8898	*P. ovale*
ldz11a	Cameroon	*P. vivax*	Ludian	*P. ovale*	4508	*P. ovale*
esy12a	Uganda	*P. vivax*	Eshan	*P. ovale*	40858	*P. ovale*
jcy13a	Ghana	*P. vivax*	Jiangchuan	*P. ovale*	1221	*P. ovale*
dld15a	Cameroon	*P. vivax*	Dali	*P. ovale*	8704	*P. ovale*
xwq17a	Guinea	*P. vivax*	Xuanwei	*P. ovale*	26258	*P. ovale*

a: Infection source identified by epidemiological investigation; b: Case confirmation by Yunnan Provincial Reference Laboratory (YNRL).
